# A Curious Case of Religious Monomania

**Published:** 1897-04

**Authors:** 


### A CURIOUS CASE OF RELIGIOUS MONOMANIA.

The *Province Medicale* for February 13th publishes the following from the *Revue Medicale*: Two young Russian girls left their homes for the purpose of accomplishing two expiatory pilgrimages, one to Notre-Dame of Lourdes, and the other to Rome. Several days afterward they arrived at the latter place, having made the entire journey on foot, without money, and living on charity. After many hardships they reached the tomb of the Apostles, where they fainted away. Here they were found and taken to the house of a pious woman, where, during a part of the night they sang interminable litanies in their own language and rendered sleep impossible to those around them.

On their trying to gain admittance to the Church of St. Peter, one of the girls became greatly enraged because the gates were closed, and it was with great difficulty that she was taken to a hospital for the insane. Two days later, her companion threw herself on her knees before the door of the vestry of the Church of St. Michael and demanded to be allowed to pass, and on the sexton's refusal she wounded him seriously by striking him on the head with a copper crucifix. She was then taken to the same hospital in which her friend was confined.—*New York Medical Journal.*

